# Unparalleled nanofibril hydrogel actuators by mimicking nature's design

**DOI:** 10.1039/d6ra02776h

**Published:** 2026-07-02

**Authors:** Farhiya Alex Sellman, Rebecca Östmans, Tobias Benselfelt

**Affiliations:** a Department of Fibre and Polymer Technology, KTH Royal Institute of Technology Stockholm 11428 Sweden fase@kth.se bense@kth.se; b Department of Fibre and Polymer Technology, Wallenberg Wood Science Center (WWSC), KTH Royal Institute of Technology Stockholm 11428 Sweden

## Abstract

Soft actuators aim to bridge the gap between rigid machines and soft matter by mimicking the flexibility and compliance of natural muscles and tissues. Stimuli-responsive hydrogels can fit this purpose through their softness and ability for large reversible shape morphing. However, the performance of hydrogel actuators is restricted by their diffusion-limited water transport, which leads to slow responses, and uniform volumetric changes that generate limited actuation forces and strains. Thus, there is a need to develop faster hydrogel actuators that can efficiently convert swelling into actuation force and strain. Anisotropic cellulose nanofibril (CNF) hydrogels offer a route to overcome these limitations by assembling charged fibrils into dense, layered sheets that are reinforced in-plane while remaining compliant in the thickness direction. This architecture redirects water uptake into uniaxial expansion or, under confinement, into high blocking pressure, thereby combining large strain, high force, and rapid response. Here, we establish how the structure and processing of charged CNF networks govern swelling-driven actuation. Specifically, we examine how fibril properties, including aspect ratio and charge density, together with sheet fabrication parameters, including drying conditions and actuator area, control the translation of water uptake into directional strain and force. Optimization of these parameters results in CNF networks that expand uniaxially by 220 times within an hour with initial strain rates of 190–300% s^−1^, reaching blocking pressures up to 4.9 MPa in less than a minute. These hydrogels are a great step towards hydrogel-based artificial muscles, which have been prevented by the slow response of previously reported stimuli-responsive hydrogels. Further development of fibrillar hydrogel actuators can lead to truly lifelike artificial muscles.

## Abstract 2

Inspired by plant cell walls, cellulose nanofibril networks redirect osmotic swelling into powerful uniaxial actuation, achieving unprecedented pressure, strain, and response rates appraoching those of lifelike artificial muscles.

## Graphical abstract

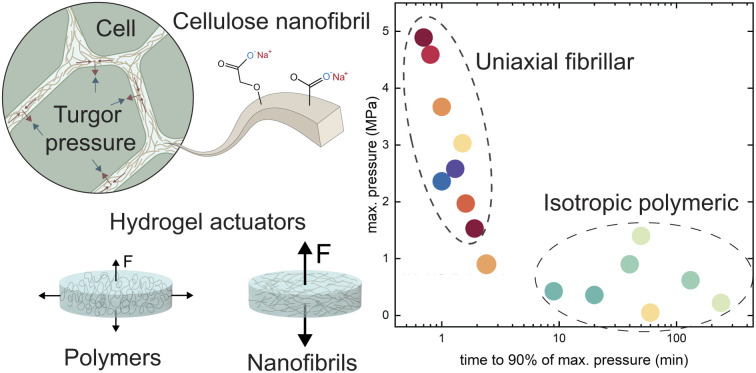

## Introduction

**Fig. 1:**
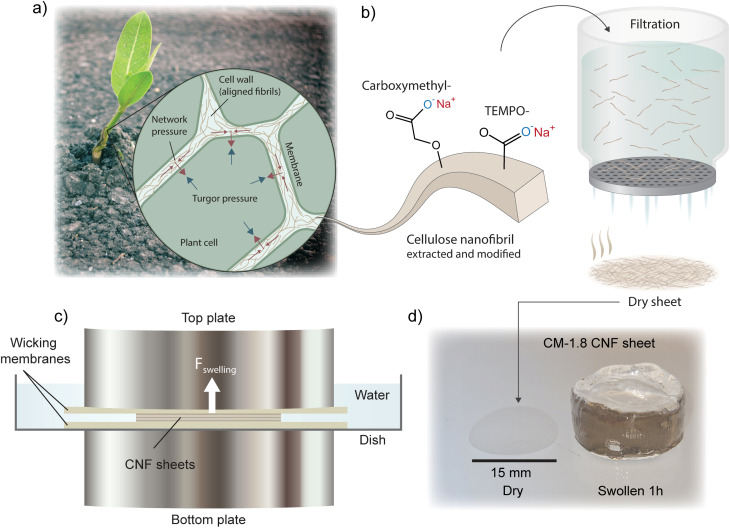
(a) Image of plants growing through asphalt, schematic magnification shows the build-up of turgor pressure in plant cells. (b) Image of the bottom-up assembly of CNF sheets by filtration. (c) Schematic that describes the characterization technique to assess actuator performance using a mechanical testing machine. (d) Picture of a CM-1.8 CNF sheet after 1 hour of swelling compared with dry initial state.

Soft actuators are highly deformable materials that can rotate, expand, contract, and bend to exhibit shape morphing in response to external stimuli.^1,2^ A key advantage of soft actuators is their lifelike mechanical properties, suitable as artificial muscles of soft robots designed for delicate tasks. Their inspiration stems from nature's efficient non-muscular movement systems; plants. Plants exhibit dynamic behaviours such as the snapping motion of the Venus flytrap or the folding of the *Mimosa pudica*.^3^ Central to many of these movements is the controlled modulation of osmotic pressure, which drives reversible changes in cell volume by transporting ions and solutes across cell membranes to create gradients that drive swelling and shrinkage.^4^ The stiffness of growing plant tissues is primarily governed by cell turgor pressure, where cellulose nanofibrils (CNFs), together with other polysaccharides and aromatic compounds, form the semipermeable cell wall that protects the cells and prevents bursting due to the osmotic pressure generated by the high concentration of molecules inside the cell.^5^ Turgor pressures as high as 0.8–0.9 MPa have been reported in onion plants.^6^ In these plants, the fibrils are organized parallel to the cell membrane, creating lateral tension that prevents cell expansion and enables plants to grow through or displace relatively hard and heavy materials by turgor presssure, as visualized in Fig. 1a.

Inspired by such natural systems, hydrogel-based actuators have emerged as materials capable of large volumetric changes in response to stimuli such as ionic strength, pH, temperature, or magnetic and electric fields.^7–11^ Their potential biocompatibility, operation in aqueous environments, and self-healing capability make them suitable for applications in biomedical settings.^12,13^ However, nature exploits hierarchical organization to achieve remarkable functions, which is difficult to replicate in uniform polymer gels.

Typically, hydrogel actuators are composed of three-dimensional covalently crosslinked polymer networks that are isotropic and undergo a uniform volumetric expansion or contraction. Consequently, many solvent-responsive hydrogels suffer from slow actuation rates due to diffusion-driven swelling and highly restrained networks,^1,14–16^ while their non-oriented deformation means that only a portion of the total strain is directional. This makes it challenging to generate both high force and large stroke. Crosslinking, required to direct the swelling pressure, further restricts the stroke.^16,17^ A solution has been to incorporate and align anisotropic nanoparticles to induce anisotropic stiffness to direct the swelling.^18^ However, these methods are complicated and may limit large-scale applicability.

An alternative strategy that has recently been explored^19^ is to fabricate the actuator from anisotropic nanoscale building blocks rather than imposing anisotropy on an initially isotropic polymer network. In such a bottom-up approach, charged CNFs dispersed in water are assembled into dense sheets by vacuum-assisted filtration (Fig. 1b) and drying. During this process, the fibrils preferentially organize in the plane of the sheet, creating a dense layered network with high in-plane reinforcement and comparatively low resistance to thickness expansion. This multi-layered structure has been visualized in several other works.^20–24^ The resulting CNF sheets are characterized by high tensile strength and stiffness, very low porosity and pronounced moisture sensitivity.^23,25–27^ Upon rehydration, the charges on the fibril surfaces generate an osmotic swelling pressure based on the difference in ion concentration between the inside of the gel and the surrounding solution, while the anisotropic network redirects the swelling primarily in the out-of-plane direction. These nanofibril hydrogels present a distinct advantage: a larger fraction of the network expansion can translate into actuation force and stroke. A further advantage is that they are simple to manufacture at scale and easy to recycle.^19^ In combination with conducting nanoparticles, they can be electrochemically controlled as electrochemical osmotic (ECO) actuators-the first hydrogel actuators with direct and precise electronic control of the hydrogel bulk.^28,29^

The aim of this work was to investigate how the fibril properties (anionic functional group, charge density and aspect ratio) influence the assembled network and actuation performance. Notably, cellulose-based materials exhibit sensitivity to moisture and liquid water, where both the water removal (drying) and uptake (swelling) processes significantly impact the final actuator performance. Hence, investigating the optimal drying conditions was important for maximising performance.

The actuators developed in this work demonstrate blocking pressures – the pressure needed to prevent swelling completely – up to 4.9 MPa within a minute, and initial directional strain rates of 190–300% s^−1^. We show that in-plane stiffness is a critical factor for achieving high actuation pressures, and that longer fibrils are advantageous for faster and higher pressure generation. Increasing the fibril surface charge increases their ability to swell and generate high actuation rates and pressures. However, above a critical threshold, excessive charge density compromises fibril contacts and reduces the anisotropic reinforcement of the hydrogel. This trade-off is particularly important for actuators with diameters below 10 mm, where a long-range internal structure plays a dominant role in limiting both in- and out-of-plane strain.

These actuators are unparalleled in the hydrogel actuator field as their actuation pressure, strain, and strain rate are orders of magnitude higher than previous reports on polymer hydrogels, even compared to the pioneering work on fibrillar hydrogel actuators.^19^ With further developments, they are probably the first hydrogel actuators with the potential to actuate at both rates and pressures suitable for lifelike artificial muscles in water-based environments.

## Results and discussion

### Nanofibrils networks, their swelling, and actuator characterization

The actuator materials are prepared from charged CNF building blocks. At the nanoscale, the individual CNFs are slender fibrils with widths of 1.6–2.7 nm and lengths of 0.44–1.6 µm, as determined by AFM (Table 1 and Fig. S1). Their high aspect ratios (*a* = l/*w*) of 200–800 enable the formation of an entangled network capable of holding large amounts of water. The volume fraction of fibrils in the network and the fibril length will govern the characteristics of the formed network, since it is related to the number of fibril-fibril contacts in the water-swollen state,^30,31^ and, consequently, the final solidity when the CNF sheets are allowed to swell to equilibrium.^32^ In a previous work, we showed that the fibril length also influences the ability of fibrils to pack during their assembly by filtration, and thereby affect the wet stiffness and strength of the CNF network.^32^

**Table 1:** The average width and length of the CNFs determined by atomic force microscopy (Fig. S1, SI) and standard deviations (*n* = 100) are given in parentheses, as well as the aspect ratio (l/*w*) calculated from these values, with error propagation provided in parentheses. Surface charge was determined by polyelectrolyte titration. A detailed characterization of CM-1.1 and CM-0.39 was provided by Östmans *et al.*^30^

Sample	Charge density, *σ* (mmol g^−1^)	Width, *w* (nm)	Length, *l* (µm)	Aspect ratio, *a*
CM-0.48	0.48	2.0 (0.6)	1.6 (1.2)	800 (640)
CM-0.53	0.53	2.6 (0.8)	0.73 (0.28)	280 (140)
TO-0.49	0.49	2.5 (0.6)	0.69 (0.27)	280 (130)
CM-1.8	1.8	2.0 (0.5)	0.72 (0.45)	360 (240)
CM-1.1	1.1	2.0 (0.7)	0.44 (0.24)	220 (140)
CM-0.39	0.39	2.7 (0.8)	0.67 (0.36)	250 (150)
CM-0.7 Holo	0.7	1.6 (0.1)	0.96 (0.06)	600(390)

A series of CNFs with surface carboxylate groups (Fig. 1b) and different charge densities were prepared. Surface functionalization was mainly achieved by carboxymethylation^33^ (CM) while one sample group was produced by TEMPO-mediated oxidation (TO) under neutral conditions.^34^Table 1 summarizes the charge densities, dimensions, and aspect ratio of the fibrils used in this study. By adjusting the reagent ratios, charge densities between 0.4 to 1.8 mmol g^−1^ were obtained, as described in the Experimental section. This enabled comparison of CNFs with different charge densities but similar aspect ratios (CM-0.39, CM-0.53, CM-1.1, CM-1.8 and TO-0.49). Additionally, CNFs with higher aspect ratios of 600 and 800 (CM-0.48 and CM-0.7 Holo) were prepared using hemicellulose-rich pulp as the starting material, the preparation procedure for these samples is reported elsewhere.^32,35^

After sheet preparation and conditioning at 50% RH and 23 °C, the moisture contents of the CNF sheets were 10.2, 10.3 and 16 wt% for CM-0.53, CM-1.1, and CM-1.8, respectively. These values are consistent with previous reports on CNF sheets, including the tendency for moisture uptake to increase with charge density.^21,36–38^

The capacity of the network of charged fibrils to swell is determined by a balance between the pressure exerted by the network resisting deformation, π_net_, and the chemical pressure driving expansion or compression. The latter can be divided into contributions from the Flory–Huggins thermodynamics of mixing, π_mix_,^39,40^ and the osmotic pressure caused by the presence of ionic groups and associated counterions, π_ion_, described by the Van't Hoff law of osmotic pressure. The total swelling pressure can therefore be written as:^41,42^

1 π_tot_ = π_net_ + π_mix_ + π_ion_

In the case of the CNF sheets, the charge density is sufficiently high, even for the lowest charge, CM-0.39, that π_ion_ is expected to dominate the swelling response, even more so at higher charge density of the fibrils.

Adding water around the sheets will initiate swelling with a pressure of π_tot_. Due to the anisotropy of the sheets, the CNF network is much stronger in-plane, so the swelling is primarily uniaxial out-of-plane. Thus, the directed swelling of these sheets allows efficient use of osmotic pressure for actuation.

The actuation performance of the fibrillar hydrogel was assessed using the force measurement setup described in Fig. 1c, utilizing a mechanical testing machine equipped with parallel plates. A Petri dish was positioned on the bottom plate and CNF sheets were placed in it, with one wicking membrane on each side of the top and bottom sheets. The role of the wicking membranes is to facilitate water accessibility to the CNF sheets. Then, the upper plate was lowered to apply a small pre-load of 1–2 N to ensure the actuator was flat. The measurement was performed by adding water and recording the blocking force (maximum π_tot_) and forces at different strains using the force sensor of the mechanical testing instrument. Generally, an arrangement of *m*-[*s*]-*m* was employed to measure the force generated, where *m* corresponds to the number of wicking membranes, and [*s*] is the number of CNF sheets forming a stack. Fig. 1d shows a CM-CNF sheet before and after one hour of swelling, resulting in in-plane and out-of-plane expansion of 20% and 21 500% and an anisotropy index of 1075.

### Blocking pressures and rate of pressure development

**Fig. 2:**
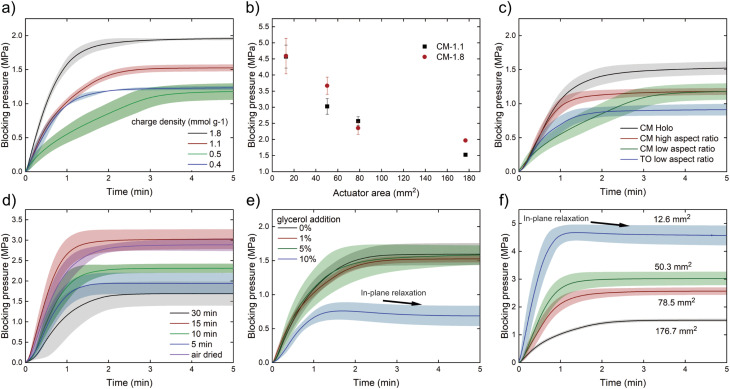
Actuation performance. (a) blocking pressure as a function of time for different charge densities, (b) comparison of blocking pressure *versus* area of CM.18 and CM1.1. Blocking pressure as a function of time for (c) different CNFs with similar charge densities (0.5–0.7 mmol g^−1^): Holo, high aspect ratio and low aspect ratio, and TO low aspect ratio, (d) drying times during sheet formation (e) glycerol content (f) actuator area of CM-1.1 sheets. Note that the samples in a, c, and e had a diameter of 15 mm, while those in d were 8 mm. The coloured regions around each curve represent the standard deviation based on 4–5 samples.

Fig. 2 presents the attained blocking pressures of CNF sheets formed from fibrils with different chemical functionalities and charge densities or differences in their drying procedure and glycerol content. A 1-[3]-1 stack order was employed to evaluate the actuation performance. Of the factors governing the swelling of the sheets (π_net_ + π_mix_ + π_ion_) in eqn (1), the charge density of the fibrils has a dominating role by inducing an ionic osmotic pressure. Increasing the charge density increases the degree of swelling and the initial swelling rate (Table 2). Higher swelling generally results in a higher pressure development rate and final blocking pressure, as displayed in Fig. 2a with differently charged CM-CNFs. The type of charged group, *i.e.*, anionic or cationic, would also affect the swelling degree of the CNF sheet and has been demonstrated to generate different actuation forces.^19^ The present work was, however, limited to anionic CNFs.

**Table 2:** Summary of the free swelling ratio after 1 hour, swelling rate in the first 10 seconds, maximum blocking pressure, and work density of highly charged CM-CNF sheets. D15 and d4 refer to the sample diameter (mm). Standard deviations are given in parentheses

Sample	Out-of-plane swelling ratio	In-plane swelling ratio	Swelling rate (% s^−1^)	Blocking pressure (MPa)	Work density (kJ m^−3^)
CM-1.8-d15	215 (17)	0.20 (0.15)	297 (13)	2.0 (0.0)	4078(986)
CM-1.8-d4	245 (45)	0.42 (0.24)	291 (18)	4.6 (0.6)	—
CM-1.1-d15	117 (7)	0.11 (0.04)	212 (40)	1.5 (0.1)	1635(429)
CM-1.1-d4	113 (4)	0.19 (0.13)	192 (3)	4.9 (0.5)	—

It should be noted, though, that a high charge density of 1.8 mmol g^−1^ (CM-1.8) compromised the in-plane reinforcing capacity of CNF networks in a way that was not found for the other CNF networks with a lower charge density. This occurred following 24 hours of water immersion, whereby the structure of the CM-1.8 hydrogel weakened, resulting in significant in-plane swelling and almost dissolution. Still, the initial uniaxial swelling allows for generating blocking pressures up to 1.9 MPa within the first minutes for samples with a diameter of 15 mm (Fig. 2a). Additionally, with the decreasing diameter of the highly charged sheets, the in-plane swelling becomes significant at even shorter times. As the areal size was reduced, the blocking pressures of CM-1.1 and CM-1.8 overlapped (Fig. 2b). Since approximately 60–67% of the cellulose chains are located at the surface of a fibril,^43^ a surface charge of 1.8 mmol g^−1^ corresponds to an effective charge density of 2.7–3 mmol g^−1^. This number is relevant when considering the solubility limit of charged cellulose like carboxymethyl cellulose (CMC), which dissolves at a degree of substitution of 0.5–1.2, equivalent to a charge density of 2.5–5 mmol g^−1^. The highly charged glucan chains on the surfaces of the fibrils are hence approaching the solubility limit. The reduced physical crosslinking and lubricating effect due to partly or fully solubilized chains probably explains the lower in-plane stiffness, similar to mixing CNFs and CMC in the initial study.^19^ Thus, CM-1.8 defines an upper charge-density limit for maximising first-cycle actuation output, but it may not be optimal for long-term or cyclic operation due to its dimensional instability upon prolonged hydration. Increased stability of the CM-1.8 hydrogel can be achieved by heat treating the CNF sheet at elevated temperatures before swelling in water, previous works have shown that heat treatment of cellulose-based materials reduce their swelling degree or even prevent disintegration.^44,45^

The fibril dimensions will also influence the performance since the characteristics of the entangled networks, such as the number of contacts per fibril, will depend on the fibril aspect ratio.^46,47^ The influence of aspect ratio on the actuator performance was investigated by preparing sheets from fibrils of different aspect ratios with the same intermediate charge (0.5–0.7 mmol g^−1^), and the results are shown in Fig. 2c. The highest blocking pressures were obtained for CM Holo and CM high aspect ratio, with aspect ratios of 600 and 800, respectively (Table 1). In these hydrogels, it is anticipated that the fibrils will wrap around each other in the dried sheet to fill all pores. This conformation is then released when water is added. Each bent fibril springs back to a more straight state and stored elastic energy (strained covalent bonds) is released, explaining the rapid and high pressure development for longer fibrils. The fibrils have a high elastic modulus, and a larger number of contacts per fibril leads to a larger stored energy per fibril and a faster pressure development as each fibril/fibril contact is released. In line with this, it was observed in our previous study^32^ that sheets containing longer fibrils exhibited a higher equilibrium swelling, *i.e.*, lower volume fractions of the wet sheets were attained. It was also distinguished that hemicellulose-rich fibrils (Holo CNFs) with a preserved fibril structure, oxidized to a charge of 0.5–0.7 mmol g^−1^, formed more swollen networks compared to similarly charged fibrils from dissolving grade fibers with lower contents of hemicellulose (4–14%). This difference indicates that if a sufficient amount of hemicellulose is present on the fibril surfaces, it could contribute to the obtained swelling pressure, which could explain the better performance of CM-Holo, as shown in Fig. 2c. Fig. 2c also shows that TO-CNF sheets reach a blocking pressure of only 0.9 MPa. The lower value could result from more damaged fibrils using this oxidation method, resulting in lower in-plane stiffness of the hydrogel network.^48^

After filtration, a CNF gel cake is obtained in which the fibrils are aligned with the plane, both their orientational alignment and as a layered structure that forms during compaction of the network upon water removal. Thus, the method of drying the gel to form a sheet will affect the water uptake capacity of the sheets as cellulosic materials are known to associate irreversibly (hornification) upon water removal,^44^ which is also a way to tune the in-plane stiffness. The data in Fig. 2d corresponds to CNF sheets subjected to different drying times at 93 °C and a reduced pressure of 95 kPa or allowed to dry under ambient laboratory conditions. As expected, the longest drying time of 30 min led to a reduced blocking pressure related to the decreased ability of the sheets to swell due to hornification.^44^ However, drying times shorter than 15 minutes were also unfavourable due to insufficient drying. After shorter drying times, the final moisture content of the sheets was higher, resulting in lower network compaction and a larger number of pores that can dissipate pressure. It is also possible that the in-plane stiffness is lower for insufficiently dried sheets as fibril contacts are not fully developed, leading to less directionality. The results also show that sheets dried for 15 min or air dried resulted in the highest blocking pressures; the latter required around 48 hours at ambient conditions. These drying procedures may result in high network compaction without severe hornification. A balanced network strength should allow enough in-plane reinforcement while not preventing out-of-plane swelling.

To test the above hypothesis, hornification effects were mitigated during the sheet formation stage by adding glycerol as a plasticizer to the CNF dispersions at different amounts. Glycerol was chosen since it has been shown to improve the redispersibility of dried CNFs, indicating that these molecules might impede close molecular contact between CNFs when water is removed.^49^ The influence of different glycerol contents on the actuation performance was investigated using a constant drying time of 15 min. Fig. 2e demonstrates that adding up to 5% glycerol to the dispersion before sheet manufacturing yielded an actuation performance similar to a sheet without glycerol. However, the presence of 10% glycerol reduced the actuation rate and notably impaired the maximum pressure generated. The pressure peak followed by a gradual pressure decline was previously also observed in polymer sheets with little in-plane reinforcement.^19^ Thus, the data suggest that glycerol indeed weakens fibril contacts upon water removal, which in turn weakens the in-plane reinforcement of the CNF network and hence does not show any advantage.

Finally, Fig. 2f shows the blocking pressure as a function of time for different actuator areas of CM-1.1 sheets. The results show that the blocking pressure increases with decreasing areal sizes, however the smallest samples also demonstrate a gradual pressure reduction after reaching maximum, indicating more in-plane relaxation. These observations indicate that there is a long-range connectivity over tens of millimetres within these materials that constrains the actuation. Reducing the sample size by cutting disrupts this connectivity, thereby relieving the constraint, but only down to a critical size beyond which the in-plane reinforcement provided by the structure is also lost.

### Force-strain relationship and work density

**Fig. 3:**
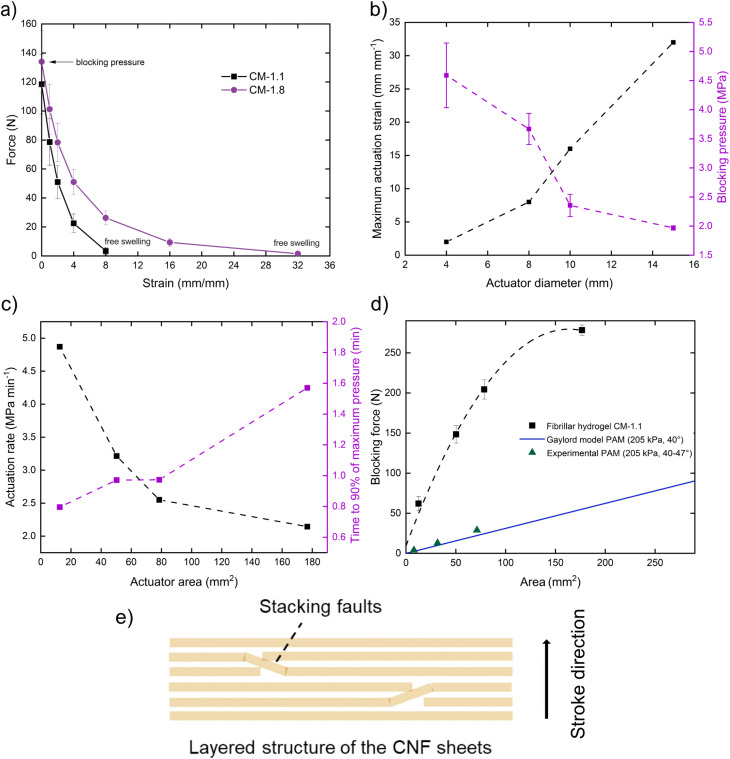
(a) Force as a function of strain for the best performing CNF samples CM-1.8 and CM1.1 with an area of 176.7 mm^2^. Note that the free swelling pressure represents the limit of the sensitivity of the instrument and that the free swelling strain is much higher. (b) maximum actuation strain and blocking pressure as a function of actuator area (c) actuation rate and time to 90% pressure for CM1.8 as a function of actuator area (d) comparison of force generation of CM-1.1 with pneumatic artificial muscle (PAM)).^50,51^ Details are given in SI. (e) Schematic of the CNF layered structure and stacking faults.

As the blocking pressure was influenced by the reinforcing effect of the network, this would be expected to be even more important during the force-strain evaluation of the hydrogels. The force as a function of strain was determined by measuring the pressure at different strains achieved by an incremental increase in the gap between the two parallel plates while the actuator was immersed in water. The strain is the ratio of the gap size to the thickness of the dry sheet, *i.e.*, the allowed swelling displacement of the sheets. Fig. 3a shows force-strain curves for the two best-performing samples in terms of blocking pressure with different charge densities. To avoid slippage between stacked sheets, which occurred as the gap size significantly increased, an arrangement of 1-[1]-0 was employed. That is, only one CNF sheet and one wicking membrane were used to minimize the reduction in blocking force caused by the compression of the wicking membranes.

The CM-1.8 hydrogel maintained a measurable force up to 3200% actuation strain, while the actuation force was nearly diminished at 800% strain for CM-1.1. The curves in Fig. 3a represent quasi–static equilibrium forces measured after stress relaxation, and at each strain the hydrogel is equilibrated for 10 minutes. Fig. S2 confirms that this equilibration time is sufficient for force stabilization, so the reported values represent stable osmotic pressures. The strain of CM-1.1 was significantly lower than CNF hydrogels shown in previous work, reaching 1600%.^19^ Due to the experimental conditions used for the preparation of these CNFs, the CM-1.1 fibrils used were relatively short and would result in a less efficient network, allowing more in-plane relaxation. Note that the maximum strain under confinement between plates is lower than the free swelling (Table 2), because the force reaches the sensitivity limit of the instrument. In the free swelling case, even a small force can over time lead to large expansion during relaxation of the nanofibril network.

An actuation strain of 3200% is almost double the previously reported limit,^19^ which was already significantly higher than polymer hydrogel actuators. Notably, actuators with smaller diameters lose in the maximum strain despite reaching higher blocking pressures, and this relationship is displayed in Fig. 3b for CM-1.8. A larger variability in maximum strain is also observed with smaller actuation areas of CM-1.8, which is likely related to the enhanced in-plane swelling of the hydrogels reported in Table 2.

The actuator area influenced many other actuator parameters. The relationship between the actuation rate or time to reach 90% of maximum pressure and the actuator area is presented in Fig. 3c. Expectedly, smaller actuators reached equilibrium swelling more quickly and had a faster response time. Herein, the time to reach 90% of maximum pressure was estimated to be ∼50 s for hydrogels with a diameter of 4 mm and 1.6 min for 15 mm hydrogels. The achieved maximum pressure also depends on the areal size of the sheets, as shown in Fig. 2f. This was previously noted to an extent for areas as small as 78.5 mm,^19^ again suggesting a structural hierarchy in the sheets that extends across centimetres.

### Unique nonlinear scaling with actuation area

Continuum mechanics models typically predict that the actuator force scales linearly with the actuation area (*F* = *σA*) resulting in a constant pressure. As shown in Fig. 3d, the CNF networks reported here do not exhibit this constant pressure-response and this may be attributed to the internal structure of the sheets. Though direct comparison is difficult, in the case of most soft actuators, the produced force scales with the square of a characteristic length *L*^2^ and is, therefore, proportional to the actuator's cross-sectional area.^52^ The actuation force as a function of the actuator diameter of the fibrillar hydrogels is compared to theoretical predictions^50^ and experimental data^51^ for a McKibben pneumatic artificial muscle (PAM) presented in Fig. 3d. The Gaylord model shows good agreement with the experimental data for PAMs and predicts a linear increase in blocking force with the square of the inner diameter. In contrast, the comparison indicates that that fibrillar hydrogel actuators generate higher actuation pressure than expected based on size and that the relationship is nonlinear. A decreasing blocking pressure with increasing areal size of the sheet is probably due to an increased network stiffness. That is, the network stiffness increases with larger actuator diameters. For a sheet between two plates with a no-slip condition, the Poisson effect, geometric constraints, and edge effects increase the apparent elastic modulus due to the restricted lateral expansion and the resulting triaxial stress state. Unfortunately, the contributions from individual effects cannot be quantified with the current measurement technique.

Another explanation for the nonlinearity is that the self-assembly *via* vacuum filtration results in a layered structure of the CNF sheets. In the internal structure, there is an interconnectivity between the layers that stabilize the structure, herein referred to as stacking faults, which are schematically depicted in Fig. 3e. We hypothesize that in large samples, boundary constraints enforce affine deformations in the out-of-plane direction, leading to a maximum contribution from the stacking faults to the stiffness. However, in smaller samples non-affine deformation, such as bending and rotation of the layers, is permitted to some extent, leading to a more compliant structure with less contribution of stacking faults to the stiffness. This hypothesis is supported by the increased in-plane swelling of smaller samples (Table 2 and Fig. S3). Future work should be dedicated at investigating this hypothesis using high resolution scattering techniques.

### Benchmarking fibrillar hydrogel actuators

**Fig. 4:**
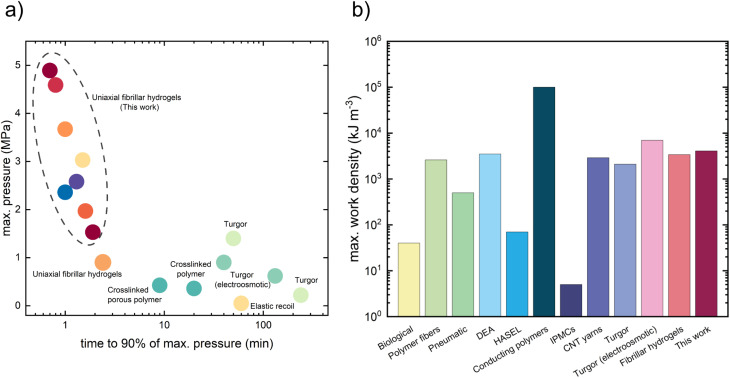
(a) Maximum pressure and time to 90% max. Pressure,^14,16,18,53–56^ and (b) maximum work density of the nanofibrillar actuators compared to other soft actuators.^57,58^ Details are given in SI.

Fig. 4a shows the actuation performance of uniaxial fibrillar hydrogels compared to other hydrogel actuators. The hydrogel actuators in this study demonstrate actuation pressure levels surpassing previous findings by over threefold and exhibit speeds to reach 90% of maximum pressure that are orders of magnitude faster than recent state-of-the-art hydrogel actuators. This makes the fibrillar hydrogels form a region in Fig. 4a resembling a straight line, where the shortest times to 90% of the pressure and maximum pressure are represented by the smallest hydrogels (4 mm in diameter). Despite the notable in-plane swelling observed with time for highly charged fibrillar networks presented in Table 2, their high directionality enables initial actuation rates of 190–300% s^−1^. The maximum work density shown in Fig. 4b is estimated from the force data in Fig. 3a plotted against the stroke. The CM-1.8 hydrogels achieved an average work density of approximately 4000 kJ m^−3^ (the best sample was 5200 kJ m^−3^), which is higher than previously demonstrated fibrillar hydrogels incorporating highly charged polyelectrolytes.^19^

**Fig. 5:**
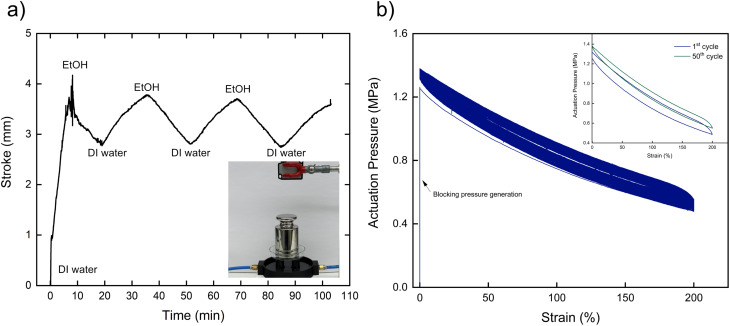
(a) Cycling with water and ethanol using four printed units with 1-[2])_4_-1 stacks of CM-1.1 CNF lifting 1 kg, inset shows measurement setup. (b) Cyclic compression of CM-1.8 with an area of 78.5 mm^2^. After initial blocking pressure generation at 0% strain, the plate separation was cyclically increased to 200% strain and returned to 0% strain while the sample remained submerged in water. The inset compares the 1st and 50th cycles, showing retained force generation and recovered blocking pressure.

To demonstrate the potential of the fibrillar hydrogel actuator, we employed a simple 3D-printed actuation unit shown in inset of Fig. 5a. The unit consisted of four hollow cylinders, open at their lower ends, into which circular CNF sheets (8 mm in diameter) were stacked in a 1-[2]_4_-1 configuration. Pistons were then inserted into the cylinders, and a 1 kg weight (pressure of 49 kPa) was placed on a Petri dish on the pillars. Actuation was initiated by flowing water through the unit. In Fig. 5a, the 1 kg load was lifted and lowered by cyclically exchanging the surrounding liquid between water and ethanol, and the corresponding displacement of the load was recorded using as laser-displacement sensor. In the first cycle, a stroke of approximately 4 mm was achieved within 8 minutes, corresponding to a strain of 540%, given that the total stack thickness was 0.74 mm including the wicking membranes. The contraction was consistent at ∼27% for the three cycles of solvent exchange, showing little indication of cyclic decay. In subsequent cycles, the expansion and contraction responses were slower, consistent with previously discussed limitations related to local liquid delivery.^19^ Notably, reliable cycling was only achieved when the initial liquid was fully removed before introducing the next solvent. The next steps to achieve high frequency lifelike actuation from fibrillar hydrogels are to (i) investigate different solvent system to swell/deswell or utilize electroosmotic stimulation, (ii) investigate a more local solvent delivery using integrated microchannels, (iii) miniaturization, and (iv) find a suitable strain range that can be cycled at high frequency.

To further evaluate the cyclic stability, which is a challenge in soft compliant materials, we performed cyclic compression measurements on CM-1.8 while the CNF sheet remained hydrated between the parallel plates. After the initial blocking pressure was measured at 0% strain, the plate separation was cyclically increased to 200% strain and returned to 0% strain for 50 cycles. The cyclic response, shown in Fig. 5b, demonstrates that the CNF hydrogel repeatedly regenerates blocking pressure without loss over 50 cycles. In fact, the pressure slightly increased after cycling, probably due to a disruption of the network upon cycling, such as loss of stacking faults that reduces π_net_. Alternatively, there is an increase in strongly bound water at fibril surfaces, exposed as the fibril networks expands during cycling, which is difficult to push out and therefore leads to increased pressure. Regardless of the exact mechanism, the impressive stability of CM-1.8, despite being the most unstable sample, further supports the robustness of these actuators.

## Conclusion

The anisotropic nanoporous network created in the CNF sheets result in actuators that overcome the challenge of slow response, while providing a span in the achieved blocking pressures, strain rates, and maximum strain depending on actuation size and network properties. In particular, highly charged CNFs (CM-1.8) can achieve strains exceeding 3000% and for larger actuation areas, CM-1.8 surpasses the rest of the investigated hydrogels in actuation performance. However, our results reveal that the optimal charge density for robust performance lies somewhat lower where the osmotic swelling does not compromise the in-plane stiffness of the hydrogel. Ideally, a high but balanced charge density should be combined with high aspect ratio fibrils to maximize both strain rate and blocking pressure while retaining the network integrity required for long-term operation. The weakening of the in-plane reinforcement of highly charged CNFs is intensified with smaller actuator sizes, which we attribute to a reduced contribution from the stabilising stacking faults within the CNF networks. As an alternative, extended drying times or heat treatments can be used to tune the consolidation of the network and, hence, its stiffness. This would induce hornification of the material and could prevent it from disintegration, but it would also decrease the swelling of the sheet to some extent depending on the heat treatment conditions. Above all, clarifying how the internal fibrillar organization depends on the sample dimensions, and how this in turn governs the mechanical response, presents an important direction for future work. A deeper understanding of these size-dependent structural effects is required to predictably engineer their actuation behaviour. With further developments in miniaturization, new organizations of fibrils, and local solvent exchange, fibrillar hydrogels hold the potential to achieve high frequency lifelike actuation.

## Experimental

### Chemicals

Peracetic acid 38–40%, monochloroacetic acid ≥99.0%, potassium phosphate monobasic (KH_2_PO4), sodium chlorite (NaClO_2_), sodium hydroxide (NaOH), glycerol ≥99.5% and 2,2,6,6-tetramethyl-1-piperidinyloxy (TEMPO, free radical), polydiallyldimethylammonium chloride 20 wt% solution (*M*_w_ 400 000–500 000), and (3-aminopropyl)triethoxysilane were purchased from Sigma-Aldrich AB (Sweden). Sodium hypochlorite (NaClO) 14%, ethanol (96% and absolute) and 2-propanol were purchased from VWR International AB (Sweden). Hydrochloric acid (HCl) 37% and methanol were purchased from Thermo Fisher Scientific. Sodium bicarbonate ≥99.7% was purchased from Honeywell Fluka. Milli-Q (Merck Milli-Q 18.2 MΩ cm^−1^) or deionized (DI) water was used throughout all experiments.

### Materials

Aditya Birla Domsjö Fabriker AB (Sweden) (dissolving grade) and Nordic Paper AB (Sweden) (hemicellulose-rich pulp) generously supplied the never-dried fully bleached sulphite pulps consisting of a mixture of Scots Pine and Norwegian spruce, while SCA Forest Products AB (Sweden) provided the spruce chips for the preparation of the specially delignified holocellulose fibers. The hemicellulose content of the pulps from Domsjö and Nordic Paper was 4% and 13–14% respectively.

### Modification of fibers

Holo fibers were obtained by a sequential mild peracetic acid (PAA) delignification of spruce chips at 85 °C, as described by Yang *et al.*^35^ The delignification was done in 5–6 rounds of PAA treatments until the fibers were liberated. Afterwards, the fibers were suspended in a NaOH solution (0.01) for 2 h, the suspension was then filtered off and washed with DI water until a conductivity below 5 µS cm^−1^.

Carboxymethylation of the fibers were performed based on an established protocol.^33^ Carboxymethylated holocellulosic nanofibrils (CM-Holo) and the high aspect ratio CM-CNF were prepared with Holo fibers or a hemicellulose-rich sulphite pulp (13–14%) as the raw material, as described previously^32^

The CM-CNF of charge densities 0.4 mmol g^−1^, 0.5 mmol g^−1^, 1.1 mmol g^−1^ and 1.8 mmol g^−1^ were prepared from a dissolving grade sulphite pulp with low residual hemicellulose and lignin. The amounts of reagents and solvent used to prepare 0.4 mmol g^−1^ and 1.1 mmol g^−1^ is described by Östmans *et al.*^30^

For the highest charge, the fibers (20 g dry mass) were liquid-exchanged in ethanol (200 ml) four times by 15 minutes each. The fibers were filtered off and drained after each liquid-exchange step. Then the fibers were impregnated for 30 minutes in a sealed container with 8.6 g of ClCH_2_COOH dissolved in 0.1 L isopropanol. 8 g of NaOH was dissolved in 0.1 L methanol and added to 0.4 L isopropanol preheated to 85 °C. The impregnated fibers were added to the heated mixture and the reaction time was set to 1 hour. After the reaction, the fibers were washed with deionized (DI) water before being suspended in a 0.01 M HCl solution for 30 minutes. Then the fibers were washed with DI water followed by immersion in a 0.01 M sodium bicarbonate solution adjusted to pH 9. Finally, the fibers were filtrated and washed with DI water until a conductivity below 5 µS cm^−1^.

2,2,6,6-tetramethyl-1-piperidinyloxy (TEMPO)-oxidation was performed on never-dried dissolving grade fibees under neutral conditions (pH 6.8) following the procedure presented by Saito *et al.*^34^ A 0.05 M phosphate buffer was preheated to 60 °C and fibers (30 g, dry mass) were added under stirring. 33.9 g sodium chlorite and 0.47 g TEMPO were added to the suspension. After dissolution, 17.6 mL of 1.7 M sodium hypochlorite was poured into the suspension to start the reaction of 2 hours. Upon completion, the suspension was filtered off and washed with DI water. The fibers were then treated with HCl and sodium bicarbonate similar to the carboxymethylated fibers.

### Preparation of CNF gels and dispersions

Except for the highly charged CM-CNF (1.8 mmol g^−1^) and CM-Holo, the chemically modified fibers were mechanically disintegrated using a high-pressure Microfluidizer M-110 EH (Microfluidics Corp., USA) by passing chambers of diameters 400/200 µm once (∼1000 bar), followed by three passages through chambers 200/100 µm (∼1650 bar) to obtain transparent CNF gels. The resulting CNF gels had a solids content of 1–1.4 wt%. The gels were diluted in MilliQ water to a concentration of 0.1–0.2 wt% by dispersing with an Ultra Turrax particle disperser (IKA, Staufen, Germany) at 12 000 rpm for 15 minutes, followed by probe sonication (VCX 750) at 80% amplitude for 10 minutes while placed in an ice batch. The dispersions were then centrifuged at 4740 g for 1 h and the supernatant was retrieved. Then, the dry content of the CNF dispersions was measured gravimetrically.

After chemical modification, the high charged CM-1.8 and the CM-Holo fibers showed an initial sufficient fibrillation allowing for a final mechanical defibrillation using a high intensity kitchen blender (Blendtech Classic 575, USA) for 5 rounds, each mixing being 50 seconds long, to obtain the CNF gels. For these fibrils, the CNF dispersions were prepared by diluting the gels and mixing with a kitchen blender for 5 rounds. The resulting dispersions contained larger aggregates which were removed by centrifugation at 4740*g* for 1 h.

### Charge density determination

A ParticleMetrix Stabino (Germany) system was used to measure the charge densities of all CNF dispersions by polyelectrolyte titration against polydiallylmethylammonium chloride. The average value of 3–4 measurements was reported.

### CNF sheet preparation

Sheets were prepared by vacuum filtration of dispersions containing 225 mg solids content CNF and 1% glycerol of the total mass, which were mixed by magnetic stirring prior to the filtration. Durapore filter membranes (Merck Millipore) with pore sizes 0.1 µm or 0.65 µm and diameter 90 mm were used. The CNF sheets were 80 mm in diameter, resulting in a grammage of 44.8 g m^−2^. After over-night filtration (∼14 h), a gel filtercake was obtained. The CNF filtercakes were either allowed to air dry at ambient conditions or subjected to drying in a Rapid Köthen sheet dryer at 93 °C, a reduced pressure of 95 kPa and for 15 min unless stated otherwise. All samples were conditioned at 50% relative humidity and 23 °C.

The moisture contents were estimated from the weights of the sheets after conditioning and those after drying at 105 °C for 4 hours as:
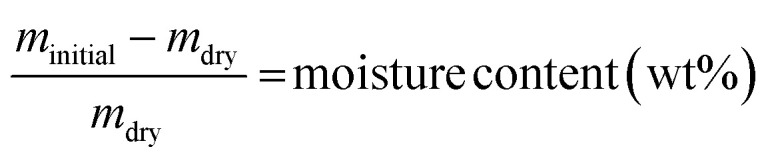


### Unrestrained swelling measurements

Circular pieces of CNF sheets measuring 15 mm in diameter were prepared using a hole puncher (inner diameter of 15 mm). These sheets were immersed in DI water for 10 seconds, 1 hour and 24 hours. The extent of their swelling was estimated by measuring the thickness change using a micrometer.

### Blocking pressure measurements

The blocking pressure was measured using an Instron 5944 with a parallel plate setup and a load cell of 500 N as shown in Fig. 1b. CNF sheets were cut into circular pieces using hole punchers of desirable diameter between 4–15 mm, giving actuator areas between 12.5–176.6 mm^2^. The CNF sheets were mounted on a polystyrene Petri dish, with Durapore membranes on top and below as wicking membranes to facilitate water transport. To ensure the stack was flat, the upper plate was lowered to apply a pressure of 1–2 N prior to start. The sample was submerged in liquid and the force recorded. The force measurements at different strains were measured by a stepwise increase in the gap size when the swelling of CNF sheets generated a steady-state force value.

Cyclic compression measurements were performed using the same parallel-plate setup. The CNF sheet the initial blocking pressure was first recorded at 0% strain. Then, the plate separation was cyclically increased to 200% strain, defined relative to the dry sheet thickness, and returned to 0% strain for 50 cycles at a strain rate of 100% min^−1^ while the sample remained submerged in water. The force was recorded continuously throughout the measurement, and the recovered blocking pressure was taken as the pressure measured after each return to 0% strain.

### Atomic force microscopy

The width and length of the CNFs were determined by AFM (Multimode 8. Bruker. Santa Barbara. USA) in ScanAsyst mode. The surface was prepared by cleaving mica and then adsorbing APTES (∼1 g L^−1^, 60 s) onto the surface. The surface was rinsed with Milli Q water and then the CNF dispersion (0.01–0.1 g L^−1^, 30–60 s) was adsorbed and then washed again with MilliQ water before it finally was dried with nitrogen gas. The thickness was determined using nanoscope analysis and the lengths were measured with image J. The average width and length were determined from analysing 100 fibrils. A detailed characterization of CM-0.39 and CM-1.1 is provided by Östmans *et al.*^30^

## Author contributions

Farhiya Alex Sellman – data curation, formal analysis, investigation, methodology, resources, validation, writing – original draft. Rebecca Östmans – data curation, formal analysis, investigation. Tobias Benselfelt – supervision, conceptualization, methodology, formal analysis, validation, data curation, visualization, writing original draft.

## Conflicts of interest

The authors have no conflict of interest to declare.

## Supplementary Material

RA-OLF-D6RA02776H-s001

## Data Availability

The data supporting this article have been included as part of the supplementary information (SI). Supplementary information is available. See DOI: https://doi.org/10.1039/d6ra02776h.
