# Supplementary material for: Unparalleled nanofibril hydrogel actuators by mimicking nature's design
Source: RSC Adv. 2026 Jul 2;16(34):31864–76. doi: 10.1039/d6ra02776h (PMC13326668; doi:10.1039/d6ra02776h)
Supplement: RA-016-D6RA02776H-s001 [file RA-016-D6RA02776H-s001.pdf]

Supplementary Information (SI) for RSC Advances.
This journal is © The Royal Society of Chemistry 2026

Unparalleled Nanofibril Hydrogel Actuators by Mimicking Nature's Design

Farhiya Alex Sellman, Rebecca Östmans, and Tobias Benselfelt**

Department of Fibre and Polymer Technology, KTH Royal Institute of Technology, Stockholm, 11428, Sweden

Department of Fibre and Polymer Technology, Wallenberg Wood Science Center (WWSC), KTH Royal Institute of Technology, Stockholm, 11428, Sweden

Corresponding author: fase@kth.se, bense@kth.se

Supporting Information

Force-Scaling Relationship

$$F = \frac{P\pi D^2}{2}(3cos^2(\theta) - 1) \tag{1}$$

Equation 1 is the Gaylord model[1] where F is the force produced by a McKibben actuator, P is the actuation pressure, D is the diameter of the PAM elastic tube at rest, and θ is the resting mesh braid angle. Values of P = 0.205 kPa and a braiding angle of 40° were used for the model, to compare to the experimental data (Figure 8) from Kothera et al.[2]

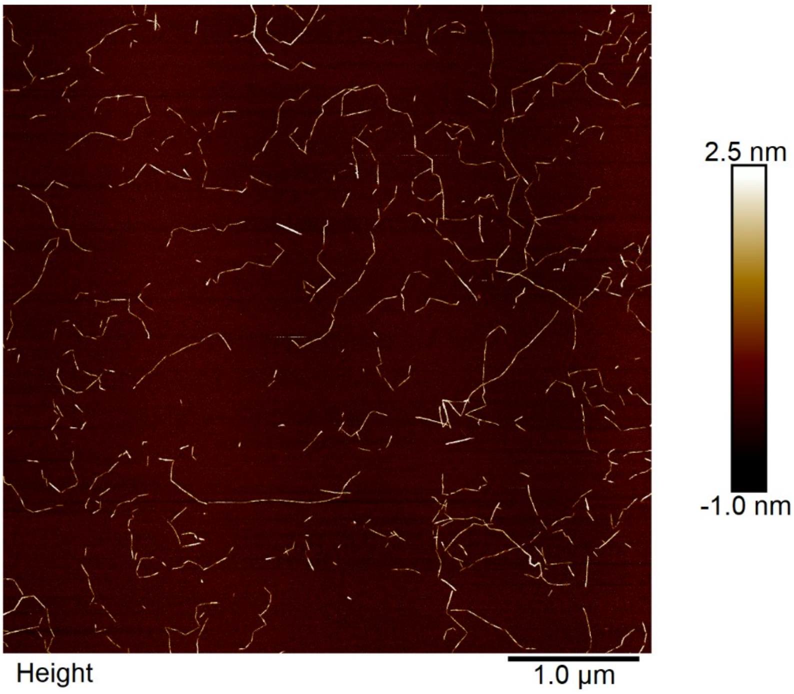

Figure S1. AFM image of CM-1.8 CNFs.

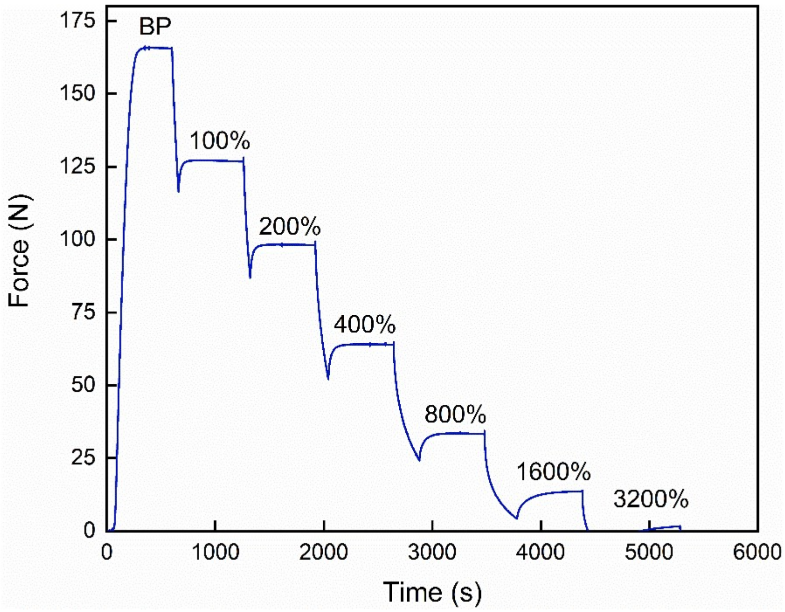

Figure S2. Force as a function of time over increasing strains of CM-1.8 CNF sample.

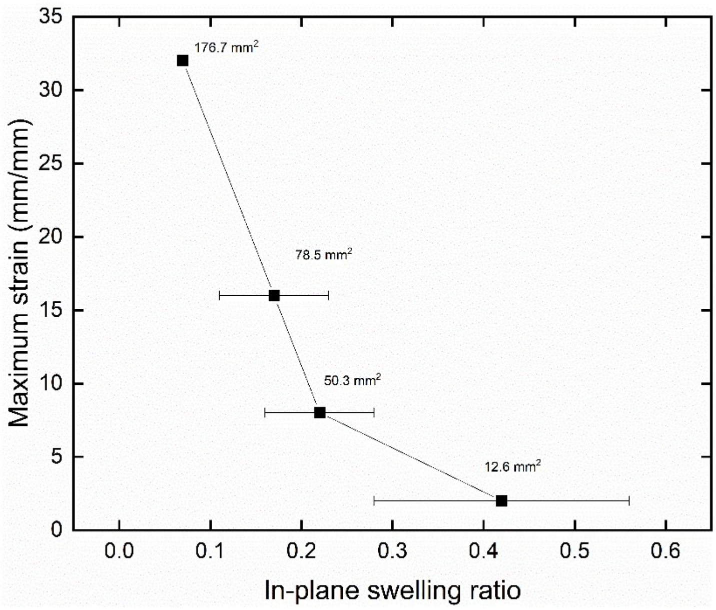

Figure S3. The in-plane swelling ratio, after 1 hour swelling, as a function of maximum actuation strain for CM-1.8 CNF.

Table S1. Literature data for actuation performance of soft actuators.

Authors	Actuator type	Pressure (MPa)	Time to 90% max pressure (min)	Source figure
This work	Uniaxial fibrillar hydrogel	0.9-4.9	0.8-2	-
Benselfelt et al.[3]	Uniaxial fibrillar hydrogel	07-0.9	1-2	3b
Na et al.[4]	Turgor	0.22-1.4	40-240	2c, 3b, & 3f
He et al.[5]	Crosslinked polymer	0.36	20	3
He et al.[5]	Porous crosslinked polymer	0.43	9	3
Ma et al.[6]	Elastic recoil	0.05	60	3b

References

(1) Lathrop, B.; Ourak, M.; Vander Poorten, E. Miniature Pneumatic Artificial Muscles for Use in Surgical Devices. In *ACTUATOR 2022; International Conference and Exhibition on New Actuator Systems and Applications*, 2022; VDE: pp 1-4.
(2) Kothera, C. S.; Jangid, M.; Sirohi, J.; Wereley, N. M. Experimental characterization and static modeling of McKibben actuators. **2009**.
(3) Benselfelt, T.; Rothemund, P.; Lee, P. S. Ultrafast, High-Strain, and Strong Uniaxial Hydrogel Actuators from Recyclable Nanofibril Networks. *Advanced Materials* **2023**, 2300487.
(4) Na, H.; Kang, Y.-W.; Park, C. S.; Jung, S.; Kim, H.-Y.; Sun, J.-Y. Hydrogel-based strong and fast actuators by electroosmotic turgor pressure. *Science* **2022**, *376* (6590), 301-307.
(5) He, X.; Zhu, J.; Yang, C. Harnessing osmotic swelling stress for robust hydrogel actuators. *Soft Matter* **2022**, *18* (28), 5177-5184.
(6) Ma, Y.; Hua, M.; Wu, S.; Du, Y.; Pei, X.; Zhu, X.; Zhou, F.; He, X. Bioinspired high-power-density strong contractile hydrogel by programmable elastic recoil. *Science advances* **2020**, *6* (47), eabd2520.
